# Synergistic Effect of a Mesothelin-Targeted ^227^Th Conjugate in Combination with DNA Damage Response Inhibitors in Ovarian Cancer Xenograft Models

**DOI:** 10.2967/jnumed.118.223701

**Published:** 2019-09

**Authors:** Katrine Wickstroem, Urs B. Hagemann, Véronique Cruciani, Antje M. Wengner, Alexander Kristian, Christine Ellingsen, Gerhard Siemeister, Roger M. Bjerke, Jenny Karlsson, Olav B. Ryan, Lars Linden, Dominik Mumberg, Karl Ziegelbauer, Alan S. Cuthbertson

**Affiliations:** 1 Thorium Conjugate Research, Bayer American Samoa, Oslo, Norway; 2 Bayer AG, TRG-Oncology II, Berlin, Germany; and; 3 Bayer AG Pharmaceuticals Division, Wuppertal, Germany

**Keywords:** monoclonal antibodies, radionuclide therapy, DNA damage response, MSLN-TTC, targeted alpha therapy, thorium-227

## Abstract

Targeted ^227^Th conjugates (TTCs) represent a new class of therapeutic radiopharmaceuticals for targeted α-therapy. They comprise the α-emitter ^227^Th complexed to a 3,2-hydroxypyridinone chelator conjugated to a tumor-targeting monoclonal antibody. The high energy and short range of the α-particles induce antitumor activity, driven by the induction of complex DNA double-strand breaks. We hypothesized that blocking the DNA damage response (DDR) pathway should further sensitize cancer cells by inhibiting DNA repair, thereby increasing the response to TTCs. **Methods:** This article reports the evaluation of the mesothelin (MSLN)-TTC conjugate (BAY 2287411) in combination with several DDR inhibitors, each of them blocking different DDR pathway enzymes. MSLN is a validated cancer target known to be overexpressed in mesothelioma, ovarian, lung, breast, and pancreatic cancer, with low expression in normal tissue. In vitro cytotoxicity experiments were performed on cancer cell lines by combining the MSLN-TTC with inhibitors of ataxia telangiectasia mutated, ataxia telangiectasia and Rad3-related (ATR), DNA-dependent protein kinase, and poly[adenosine diphosphate ribose] polymerase (PARP) 1/2. Further, we evaluated the antitumor efficacy of the MSLN-TTC in combination with DDR inhibitors in human ovarian cancer xenograft models. **Results:** Synergistic activity was observed in vitro for all tested inhibitors (inhibitors are denoted herein by the suffix “i”) when combined with MSLN-TTC. ATRi and PARPi appeared to induce the strongest increase in potency. Further, in vivo antitumor efficacy of the MSLN-TTC in combination with ATRi or PARPi was investigated in the OVCAR-3 and OVCAR-8 xenograft models in nude mice, demonstrating synergistic antitumor activity for the ATRi combination at doses demonstrated to be nonefficacious when administered as monotherapy. **Conclusion:** The presented data support the mechanism-based rationale for combining the MSLN-TTC with DDR inhibitors as new treatment strategies in MSLN-positive ovarian cancer.

Targeted α-therapy represents a promising modality whereby the high energy (5–9 MeV) and short range (50–100 μm) of the α-particle can be exploited together with a targeting moiety to specifically irradiate a tumor while sparing normal tissue (1,2). Targeted ^227^Th conjugates (TTCs) comprise the α-particle emitter ^227^Th complexed to a 3,2-hydroxypyridinone chelator covalently attached to a tumor-targeting antibody (3–6). ^227^Th has a half-life of 18.7 d, and the decay chain deposits 5 high-energy α- and 2 β-particles (7). The dense ionization track of the α-particle induces complex DNA damage both directly and indirectly via generation of reactive oxygen species (1,8). In this study, we explore treatment strategies using a mesothelin (MSLN)-TTC conjugate (BAY 2287411) in combination with DNA damage response (DDR) inhibitors. MSLN is a 40-kDa membrane-anchored glycoprotein that is frequently overexpressed in cancer types including mesothelioma, ovarian, lung, and pancreatic cancer (9). In contrast, MSLN has limited expression in nonmalignant tissue and is therefore considered a suitable antigen for targeted therapies (10–12). We have previously demonstrated that MSLN-TTC induces specific antitumor activity. This agent is currently in clinical phase I indicated for mesothelioma and ovarian cancer (NCT03507452).

Efficient DNA damage repair is a contributing factor to poor prognosis and treatment outcome after radiotherapy (13,14). We therefore hypothesized that DDR pathways also contribute to efficacy after treatment with MSLN-TTC. This study investigated in vitro and in vivo combinations of inhibitors (inhibitors are denoted herein by the suffix “i”) of the key mediators of DDR, including ataxia telangiectasia mutated (ATM), ataxia telangiectasia and Rad3-related (ATR), DNA-dependent protein kinase (PK), and poly[adenosine diphosphate ribose] polymerase (PARP) 1/2 (15) with MSLN-TTC.

## MATERIALS AND METHODS

### Cells

OVCAR-3 and OVCAR-8 were from American Type Culture Collection, NCI-H226 was from the National Institutes of Health, and Capan-2 and HT29 were from Deutsche Sammlung von Microorganismen und Zellkulturen GmbH. All cell lines were authenticated using polymerase chain reaction fingerprinting. Cells were maintained at 37°C, 5% CO_2_. Capan-2 and NCI-H226 were cultured in RPMI medium, HT29-MSLN in RPMI with 1% sodium bicarbonate and 2% hygromycin, OVCAR-3 in RPMI with 10 μg/mL insulin (bovine) and 2 mmol/L glutamine, and OVCAR-8 in DMEM/F12. Culture medium was supplemented with 10% fetal bovine serum and 1% penicillin/streptomycin for all cell lines. HT29 cell transfection with human MSLN was as described previously (10).

### Compounds

BAY 1895344 (2-[(3*R*)-3-methylmorpholin-4-yl]-4-(1-methyl-1H-pyrazol-5-yl)-8-(1H-pyrazol-5-yl)-1,7-naphthyridine) (WO2016020320), PARPi (olaparib), ATMi (AZD0156), and DNA-PKi (VX-984) were synthesized at Bayer AG.

### Preparation of the MSLN-TTC

The 3,2-hydroxypyridinone chelator was conjugated to the MSLN and isotype control antibodies and labeled with ^227^Th as previously described (4,16). Antibody-chelator conjugate formulations were incubated with 0.5–2.5 MBq of ^227^Th at room temperature for 60 min. Radiochemical purity, defined as the amount of ^227^Th bound to the MSLN-TTC, was determined by instant thin-layer chromatography.

### In Vitro Experiments

OVCAR-3 cells were seeded in 12-well (2 mL/well) or 6-well (4 mL/well) plates 24 h before treatment with MSLN-TTC (1/10 kBq/mL) and ATRi (10 nM) or PARPi (0.5 μM). Because of the half-life of TTC (18.7 d), the cells were exposed to treatment for 3 d before fixation with 70% ethanol, followed by staining and detection with Guava Easycyte 8HT. Cell cycle analysis was determined by staining with propidium iodide/RNase (catalog number F10979; Thermo Fisher), double-strand breaks (DSBs) with γH2A.X antibody (catalog number 9720, Alexa Fluor-647 conjugate; Cell Signaling Technology), and apoptotic cells with cleaved caspase-3 antibody (catalog number 9669, Alexa Fluor-488 conjugate; Cell Signaling Technology). The γH2A.X-positive/cleaved caspase-3–negative cells were collected for the determination of DSBs, in order to exclude apoptotic DNA cleavage from the measurements. Viability was determined after 5 d of exposure by CellTiter Glo 2.0 (catalog number G9243; Promega). Data were analyzed using FlowJo software (version 10) and GraphPad Prism software (version 7).

Combination experiments were conducted in 384-well plates (30 μL/well, 30,000 cells/mL for Capan-2, NCI-H226, and OVCAR-3; 100,000 cells/mL for HT29-MSLN). After 24 h, cells were treated with a titration of MSLN-TTC and DDRi using a D300e digital dispenser (Tecan) in the following combination ratios with MSLN-TTC (C1) and the DDRi (C2): 1 × C1, 0.9 × C1 + 0.1 × C2, 0.8 × C1 + 0.2 × C2, 0.7 × C1 + 0.3 × C2, 0.6 × C1 + 0.4 × C2, 0.5 × C1 + 0.5 × C2, 0.4 × C1 + 0.6 × C2, 0.3 × C1 + 0.7 × C2, 0.2 × C1 + 0.8 × C2, 0.1 × C1 + 0.9 × C2 and 1 × C2. MSLN-TTC was titrated in the range 0.001–5 kBq/mL for HT-29-MSLN and 0.01–50 kBq/mL for Capan-2, OVCAR-3/8, and NCI-H226, at a specific activity of 40 kBq of ^227^Th/μg of antibody chelator conjugate for HT29-MSLN, Capan-2, and OVCAR-3/8 and 20 kBq of ^227^Th/μg for NCI-H226. DDR inhibitors were dissolved in dimethyl sulfoxide and titrated in the range 0.002–10 μM for ATRi (BAY 1895344), ATMi (AZD0156), or DNA-PKi (VX-984) and 0.01–50 μM for PARPi (olaparib/AZD2281). After incubation (HT29-MSLN: 5 d; OVCAR-3/8, NCI-H226, and Capan-2: 7 d), viability was determined using CellTiter Glo according to the manufacturer’s protocol. Optimization for each cell line was required to account for variations in doubling time, receptor density, internalization rates, and radiosensitivity. As a result, specific activity, total activity, and incubation time vary among the cell lines. Half-maximal inhibitory concentration isobolograms were generated by plotting half-maximal inhibitory concentrations from MSLN-TTC/DDRi on the *x*- and *y*-axes, respectively. The combination index (CI) was determined according to the median-effect model of Chou (17), with CI < 0.8 defined as synergistic effect, 0.8 ≤ CI ≤ 1.2 defined as additive effect, and CI > 1.2 defined as antagonistic effect.

### In Vivo Efficacy

Experimental protocols were approved by the National Animal Research Authority and conducted according to the recommendations of the Federation of European Laboratory Animal Science Association and Directive 2010/63/EU of the European Parliament regulations. All animals received an intraperitoneal injection of murine IgG2a antibody (200 μg/animal; UPC10; Sigma) 16–24 h before treatment to block nonspecific spleen uptake (18). The OVCAR-3–bearing mice were supplemented with 17-β-estradiol either in drinking water (4 mg/L, average daily dose of 1 mg/kg [Sigma-Aldrich], catalog number E8875) or subcutaneous implantation of pellets (1.7 mg/pellet, timed release: 90 d [Innovative Research of America], catalog number SE-121). The tumor growth and the body weights were measured by caliper every second or third day. Animals were sacrificed by cervical dislocation on reaching the humane endpoint (tumor volume ≥ 1,500 mm^3^; body weight loss ≥ 20%).

Five million OVCAR-3 cells in 0.1 mL of phosphate-buffered saline were inoculated subcutaneously into mice (female, 4–6 wk old, HsdCpb: athymic nude Foxn1^nu^; Department for Comparative Medicine, Oslo, Norway; first generation of animals obtained from Harlan, Amsterdam, The Netherlands). At an average tumor area of 25–35 mm^2^, the mice (*n* = 10) received a single intravenous injection of MSLN-TTC (100, 250, or 500 kBq/kg, 0.14 mg/kg), isotype control (250 kBq/kg, 0.14 mg/kg), nonradioactive MSLN antibody-chelator conjugate (0.14 mg/kg), or vehicle. One group was treated with MSLN-TTC (100 kBq/kg, 0.14 mg/kg) and ATRi (40 mg/kg in 60% polyethylene glycol 400/10% ethanol/30% water, dosed twice daily, 3 d on/4 d off, 4 wk) or PARPi (50 mg/kg in phosphate-buffered saline supplemented with 10% 2-hydroxylpropyl-β-cyclodextrin, daily, 4 wk). At the study endpoint, tumors treated with MSLN-TTC (500 kBq/kg) and vehicle groups were stained for γH2A.X using rabbit anti-γH2A.X antibody (MABE205; Millipore) with BrightVision rabbit/HRP (DPVR110HRP; Immunologic) incubated for 30 min followed by 3,3′-diaminobenzidine for 5 min.

Three million OVCAR-8 cells in 0.1 mL of Matrigel were inoculated subcutaneously into mice (female, 4–6 wk old, CB-17/lcr-Prkdc^scid^ mice; Janvier). At an average tumor area of 30–40 mm^2^, the mice (*n* = 10) received 3 intravenous injections of MSLN-TTC (200 kBq/kg, 0.14 mg/kg, days 1, 22, and 43) and ATRi (40 mg/kg, dosed twice daily, 2 d on/5 d off, 7 wk). Blood samples were collected at the endpoint of the study and analyzed with Hemavet (HV950; Drew Scientific, Inc.).

To evaluate the cooperativity of combination treatment, the expected additivity was calculated according to the Bliss model (19): C = A + B − A × B; wherein C is the expected treatment-to-control ratio of the combination of drug A and drug B if they act additively, A is the treatment-to-control ratio of drug A, and B is the treatment-to-control ratio of drug B. An excess of more than 10% over the expected additive effect is assumed to indicate synergism, and an excess of less than 10% of the expected additive effect is assumed to indicate antagonism.

### Statistics

Statistical significance was evaluated using GraphPad Prism software (version 7.0) applying the Student *t* test and 1-way ANOVA followed by the Tukey test.

## RESULTS

### Preparation and Characterization of MSLN-TTC

Radiolabeling was effected by incubation of ^227^Th with the antibody conjugate at ambient temperatures (3,4,6). The radiochemical purity was determined by instant thin-layer chromatography for each experiment and was consistently at least 95%. The binding affinity was not impaired by conjugation or radiolabeling (Supplemental Figs. 1 and 2; supplemental materials are available at http://jnm.snmjournals.org).

### Synergistic Effect of MSLN-TTC and DDR Inhibitors In Vitro

**FIGURE 1.**
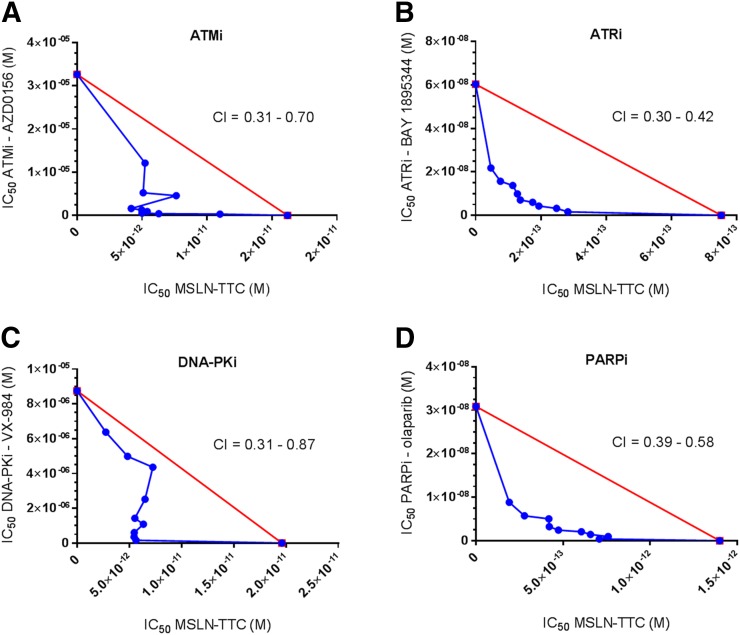
Synergistic effect of MSLN-TTC and DDRi on ovarian cancer cell line OVCAR-3. Half-maximal inhibitory concentration (IC_50_)-isobolograms with MSLN-TTC and ATMi AZD0156 (A), ATRi BAY 1895344 (B), DNA-PKi VX-984 (C), and PARPi olaparib (D) on OVCAR-3 cells. CI (mean, *n* = 3) was determined according to median-effect model of Chou (17), with CI < 0.8 defined as synergistic effect, 0.8 < CI > 1.2 defined as additive effect, and CI > 1.2 defined as antagonistic effect.

In vitro cytotoxicity experiments were performed on cell lines of different tissue origin and MSLN expression (Table 1). The effect of the combination was evaluated by isobolograms as exemplified in Figure 1 for the OVCAR-3 cell line and Supplemental Figures 3–6. Data analysis according to the median-effect model of Chou gave combination indices indicating synergistic, additive, and antagonistic effects (17). All DDR inhibitors demonstrated synergy in combination with MSLN-TTC in the OVCAR-3 cell line (Table 1). Furthermore, the ATRi induced the strongest synergy in combination with MSLN-TTC in all tested cell lines. In contrast, the synergistic effect was not as well pronounced for ATMi or DNA-PKi, which induced both additive and antagonistic effects.

**TABLE 1:** In Vitro Characterization of MSLN-TTC and DDRi

Cancer type	Antibodies bound per cell	Monotreatment MSLN-TTC, IC_50_ (kBq/mL)	Monotreatment DDRi, IC_50_ (nM)				CI			
			ATMi	ATRi	DNA-PKi	PARPi	+ATMi*	+ATRi*	+DNA-PKi*	+PARPi*
NCI-H226, lung/mesothelioma	2,000	2.0	0.2	10	90	200	0.9 ± 0.2^(add)^	0.5 ± 0.1^(s)^	0.7 ± 0.1^(s)^	0.8 ± 0.1^(add)^
Capan-2, pancreatic	20,000	28	40	70	16	19	1.1 ± 0.1^(add)^	0.6 ± 0.2^(s)^	1.2 ± 0.1^(add)^	1.0 ± 0.1^(add)^
OVCAR-3, ovarian	60,000	2.5	0.3	60	90	1.4	0.7 ± 0.1^(s)^	0.4 ± 0.04^(s)^	0.7 ± 0.2^(s)^	0.5 ± 0.1^(s)^
OVCAR-8, ovarian	40,000	3.0	ND	120	ND	ND	ND	0.7 ± 0.1^(s)^	ND	ND
HT29-MSLN, colorectal	240,000	0.2	400	60	30	200	1.4 ± 0.3^(ant)^	0.7 ± 0.1^(s)^	0.9 ± 0.4^(add)^	0.6 ± 0.1^(s)^

* In combination with MSLN-TTC. ^(add)^ = additive, ^(ant)^ = antagonistic, ^(s)^ = synergistic.

IC50 = half maximal inhibitory concentration; ND = not determined.

CI data are mean ± SD (*n* = 3).

### ATRi and PARPi Potentiate MSLN-TTC Activity by Suppressing DNA Damage Repair

On the basis of the robust synergy observed in vitro, we investigated the mechanism of combination treatment in the OVCAR-3 model to determine whether the enhanced effect correlated with inhibition of DNA damage repair.

**FIGURE 2.**
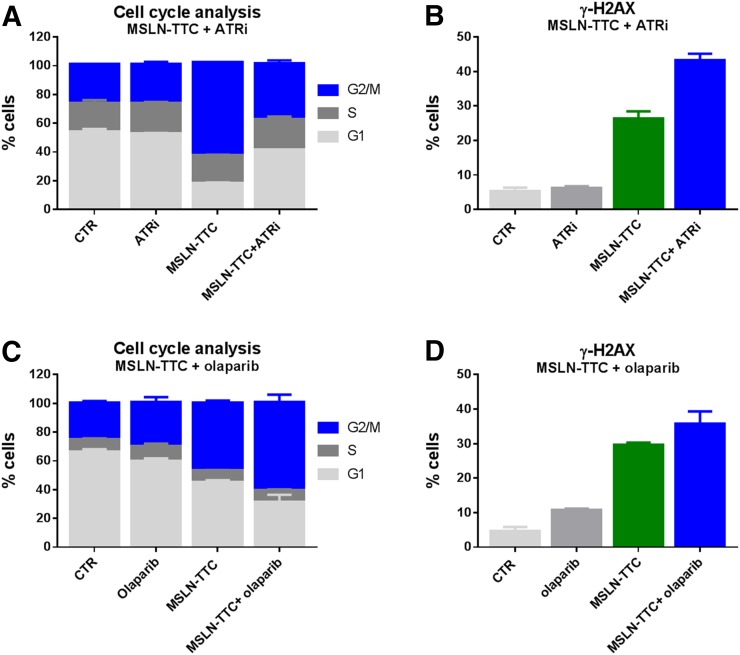
In vitro mechanistic experiments from MSLN-TTC ± ATRi BAY 1895344 or PARPi olaparib on OVCAR-3. (A and B) Cell cycle analysis (A) and γ-H2A.X (B) determined after treatment with MSLN-TTC (10 kBq/mL) and ATRi (10 nM) for 3 d. (C and D) Cell cycle analysis (C) and γ-H2A.X (D) determined after treatment with MSLN-TTC (1 kBq/mL) and PARPi (0.5 μM) for 3 d. Error bars represent SD of mean, *n* = 3.

DSBs and cell cycle arrest in G2 or M phase was observed as evidenced by increased γH2A.X and accumulation of cells with 4N DNA content, respectively, in cells treated with a single sublethal dose of MSLN-TTC. When combined with a nonefficacious dose of ATRi (10 nM), the percentage of cells entering the G2/M cell cycle phase was reduced. In contrast, the number of γH2A.X-positive cells increased (Figs. 2A and 2B; Supplemental Table 1), correlating with a higher level of apoptosis and a decrease in cell viability, demonstrating the potency of the combination (Supplemental Figs. 7A and 7B; Supplemental Table 1). When MSLN-TTC was combined with a nonefficacious dose of PARPi (0.5 μM), we observed an increase in cell cycle arrest and γH2A.X (Figs. 2C and 2D; Supplemental Table 2). This translated to a modest increase in potency of MSLN-TTC as determined by a viability assay and an increase in apoptosis (Supplemental Figs. 7C and 7D; Supplemental Table 2), although to a lesser extent than in the combination with ATRi.

### Tumor Growth Inhibition of MSLN-TTC in the OVCAR-3 Ovarian Cancer Xenograft Model

**FIGURE 3.**
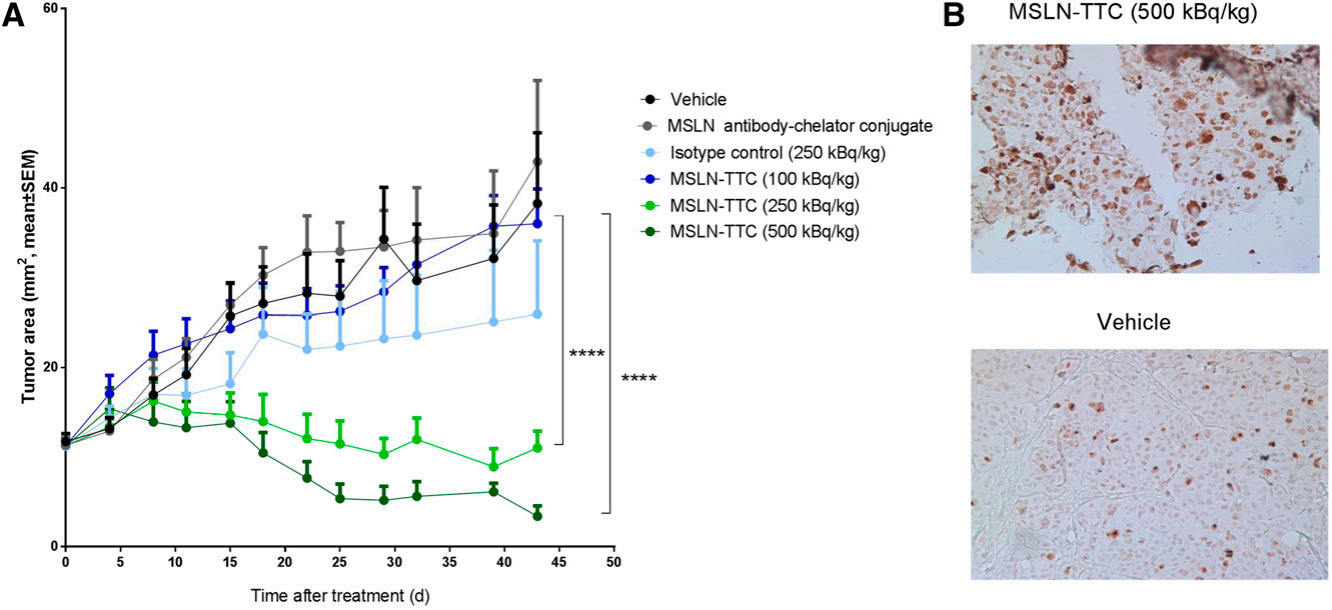
In vivo characterization of monotreatment with MSLN-TTC in OVCAR-3 xenograft model. (A) Tumor size determined after single dose (100, 250, or 500 kBq/kg, 0.14 mg/kg, intravenous) of MSLN-TTC, isotype control (250 kBq/kg, 0.14 mg/kg, intravenous), or vehicle control. Statistical analysis was performed using 1-way ANOVA followed by Tukey test. ****P < 0.0001. (B) Immunohistochemistry of γH2A.X on tumors after single dose (500 kBq/kg, 0.14 mg/kg, intravenous) of MSLN-TTC or vehicle control.

The in vivo efficacy was determined by measuring changes in tumor area after administration of a single dose of MSLN-TTC at 100, 250, and 500 kBq/kg. An isotype control (250 kBq/kg) was included for comparison. Statistically significant tumor growth inhibition compared with vehicle was achieved for the MSLN-TTC at 250 and 500 kBq/kg (*P* < 0.0001), with the higher dose resulting in a more pronounced reduction in tumor size (Fig. 3A). No statistical significance was observed for the dose of 100 kBq/kg of MSLN-TTC or for the isotype control versus the vehicle control group. Immunohistochemistry analysis of γH2A.X on tumor tissue demonstrated induction of an increased level of DSBs compared with vehicle control after treatment with MSLN-TTC (500 kBq/kg) (Fig. 3B).

### Enhanced Potency of MSLN-TTC in Combination with ATRi or PARPi in OVCAR-3 and OVCAR-8

On the basis of the observed synergistic effect of MSLN-TTC in combination with ATRi and PARPi in vitro, we further evaluated the in vivo efficacy of both combinations in an OVCAR-3 xenograft model. Since a single dose of 250 kBq/kg was highly efficacious as a monotherapy, we selected the lower dose of 100 kBq/kg for the combination study. ATRi was dosed 1 wk after MSLN-TTC at 40 mg/kg dosed twice daily, 3 d on/4 d off, for 4 wk and PARPi at 50 mg/kg daily for 4 wk based on internal and published data, respectively (20). Results from biodistribution studies show that TTCs typically accumulate in tumors over 4–7 d and have extensive retention (4,5). Given the 18.7-d half-life of ^227^Th, the absorbed dose to tumor is delivered over several weeks. Thus, DDR inhibitors are administered over a 4-wk period as more DNA damage is induced over time by the TTC.

**FIGURE 4.**
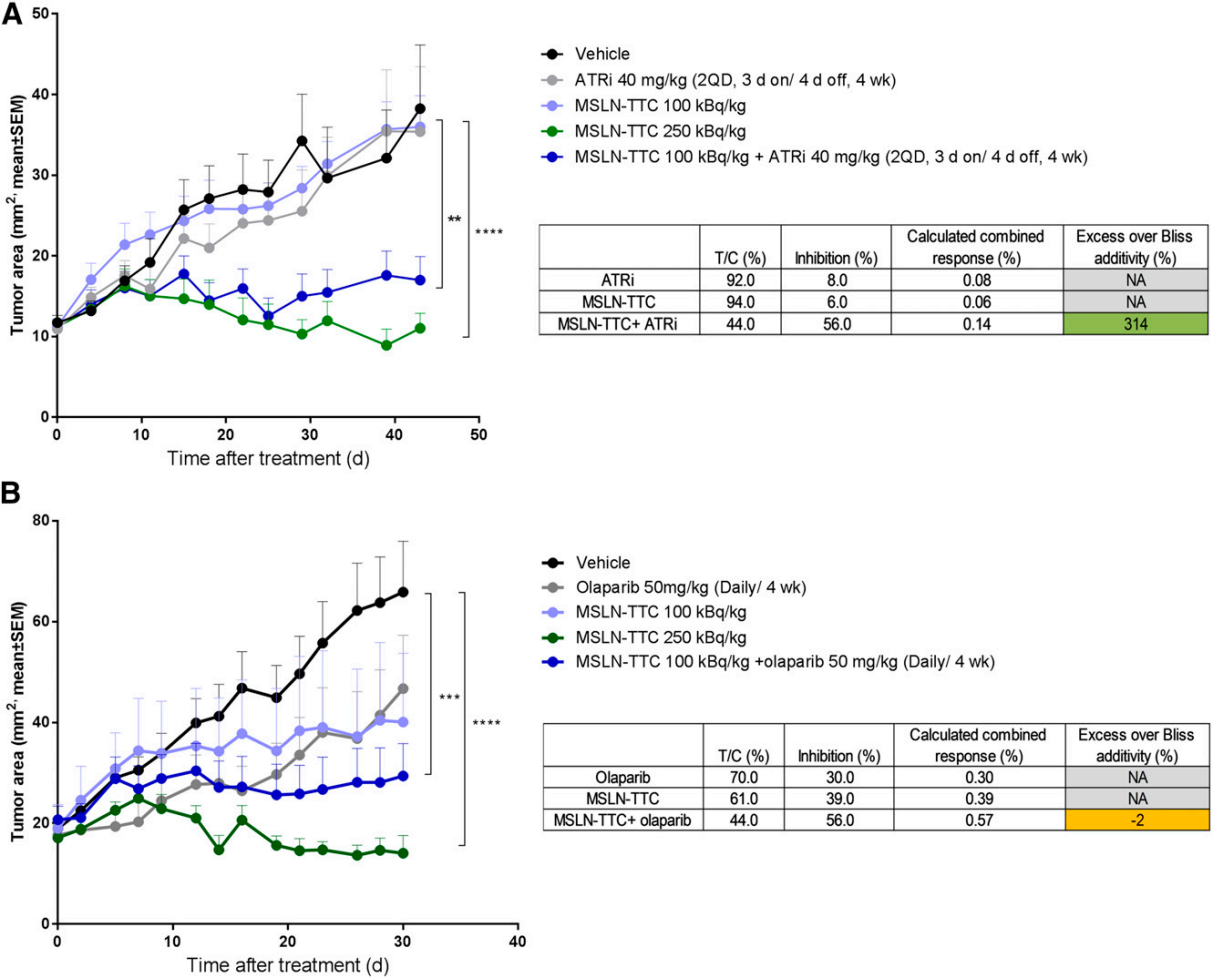
In vivo efficacy of MSLN-TTC in combination with ATRi or PARPi in OVCAR-3 xenograft model. (A) Tumor size determined after single dose of MSLN-TTC (100 kBq/kg, 0.14 mg/kg, intravenous) and ATRi (40 mg/kg dosed twice daily, 3 d on/4 d off, 4 wk). (B) Tumor size determined after single dose of MSLN-TTC (100 kBq/kg, 0.14 mg/kg, intravenous) and PARPi (50 mg/kg once daily, 4 wk). Statistical analysis was performed using 1-way ANOVA followed by Tukey test. **P < 0.01. ***P < 0.001. ****P < 0.0001. T/C = treatment-to-control ratio.

The ATRi combination enhanced the potency of the MSLN-TTC, with significant tumor growth inhibition compared with the vehicle control group (*P* < 0.0001) and the monotherapy group (*P* < 0.01) (Fig. 4A). Furthermore, the combination effect was determined to be synergistic based on the Bliss model (19). Although the PARPi combination was efficacious compared with the vehicle group, significance was not achieved when compared with the respective MSLN-TTC monotherapy; the combination effect was determined to be additive (Fig. 4B). Both dosing schedules were well tolerated as evidenced by the stable body weights in all groups tested (Supplemental Fig. 8).

**FIGURE 5.**
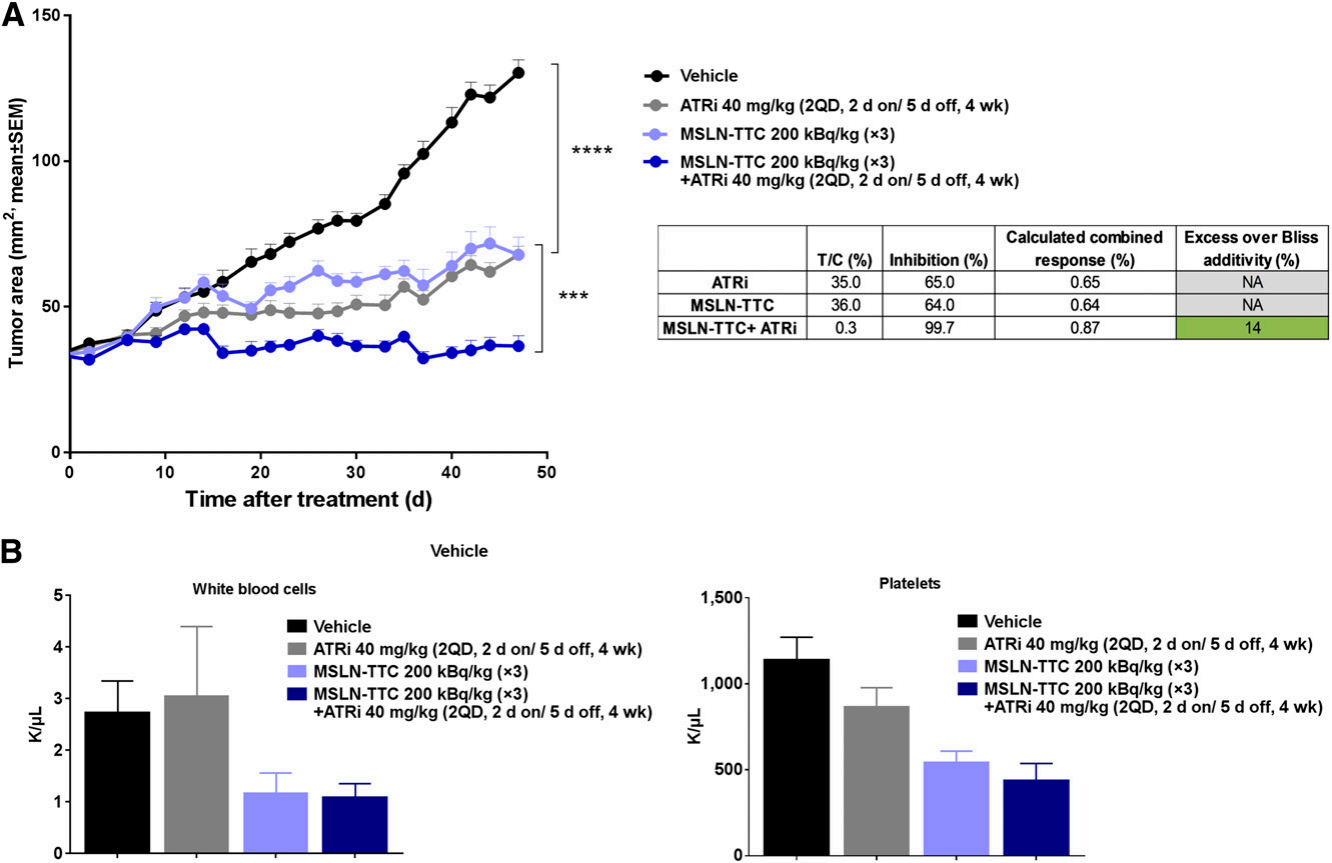
In vivo efficacy of MSLN-TTC in combination with ATRi in OVCAR-8 xenograft model. (A and B) Tumor size (A) and white blood cells and platelets (B) determined at endpoint of study after 3 (intravenous) injections of MSLN-TTC (200 kBq/kg, 0.14 mg/kg) and ATRi (40 mg/kg dosed twice daily, 2 d on/5 d off). Statistical analysis was performed using 1-way ANOVA followed by Tukey test. ***P < 0.001. ****P < 0.0001. T/C = treatment-to-control ratio.

We further explored the combination of MSLN-TTC and ATRi in the OVCAR-8 ovarian cancer xenograft model, which had previously demonstrated in vitro synergy from the ATRi combination (Supplemental Fig. 6). Because of a lower receptor level and a more rapid growth rate, the dosing schedule was changed to 3 × 200 kBq/kg MSLN-TTC and ATRi (40 mg/kg, dosed twice daily, 2 d on/5 d off). There was a significant effect of monotherapy with MSLN-TTC or ATRi compared with vehicle-treated control in the OVCAR-8 model (*P* < 0.0001). The combination induced a significantly enhanced antitumor effect as compared with the monotherapy (*P* < 0.001) (Fig. 5A), which was synergistic. No significant change in body weight was observed in any of the treatment groups (Supplemental Fig. 8). However, a significant reduction in white blood cells (*P* < 0.001) and platelets (*P* < 0.001) was observed for MSLN-TTC monotherapy and the ATRi combination (Fig. 5B) in comparison to vehicle control. The reduction in white blood cells and platelets was comparable for MSLN-TTC and the combination, indicating no combined-toxicity effect at the time point evaluated.

## DISCUSSION

Several clinical and preclinical studies support the synergistic effects of combining targeted drugs with radiation therapy for the treatment of solid tumors (21). Drug combinations have the potential to sensitize tumor cells to ionizing radiation, enhancing the therapeutic effect while minimizing side effects and damage to normal tissue. We report herein the preclinical evaluation of a combination of inhibitors of DNA damage repair with systemic targeted α-therapy. As the mode of action of targeted α-therapy is based on the induction of complex DNA damage, we postulated that combinations with DDR inhibitors may induce a synergistic effect. α-particles are considered to be highly cytotoxic because of extensive induction of DSBs, and the development of radio-resistance has not been reported for α-particle therapy (22,23). In contrast, low-linear-energy transfer particles or rays induce sublethal damage such as single-strand breaks, which cells have a higher capacity to repair by, for example, excision repair mechanisms (24). In the present study, a MSLN-specific antibody-chelator conjugate was prepared using the fully human anti-MSLN antibody anetumab. The antibody binds specifically to MSLN with high affinity (dissociation constant, 14 nM) and induces efficient internalization on MSLN expressing cell lines.

We initially screened for synergistic effects of MSLN-TTC and DDR inhibitors in viability assays. The MSLN-TTC was shown to synergize with all DDR inhibitors tested (ATMi, ATRi, DNA-PKi, and PARPi), whereas a more pronounced effect was observed for ATRi and PARPi. Mechanistic studies evaluating the ATRi combination on OVCAR-3 cells revealed a significantly higher proportion of cells continuing through the cell cycle into G1/S phase than for the MSLN-TTC monotherapy (*P* < 0.0001). The reduced level of cell cycle arrest for the ATRi combination can be explained by the blockage of the ATR kinase at the G2 cell cycle checkpoint (25). It also appeared that this combination resulted in a higher level of DSBs as evidenced by γ-H2A.X staining. Furthermore, the potency of the combination was reflected in an increase in apoptosis and reduction in cell viability. In contrast, the PARPi combination appeared to increase cell cycle arrest, and γ-H2A.X staining was less pronounced, as was induction of apoptosis markers and cell viability. The cell cycle data therefore appear to reflect key mechanistic differences between ATR, involved in cell cycle arrest and DNA DSB repair, and PARP, involved in single-strand repair.

Ovarian cancer is an indication with an unmet medical need and is a preferred indication for the MSLN-TTC clinical study. On the basis of the strong in vitro synergy on the ovarian cancer cell line OVCAR-3, we chose this model for in vivo evaluation of the combinations of MSLN-TTC with the PARPi and the ATRi. First, a dose response study was performed in OVCAR-3 tumor–bearing mice treated with a single dose of 100, 250, or 500 kBq/kg of MSLN-TTC. Significant inhibition of tumor growth was measured at 250 and 500 kBq/kg, whereas 100 kBq/kg gave no measurable effect on growth inhibition compared with vehicle control. Nonsignificant efficacy was observed for the isotype control at 250 kBq/kg, as is likely to arise from a non–target-mediated enhanced permeability and retention effect (26). Immunohistochemical analysis of γH2A.X indicated higher levels of DSBs in the 500 kBq/kg group than in the vehicle controls, further supporting the validity of testing the DDR inhibitor combination in this model at lower doses.

When MSLN-TTC was combined with the PARPi at a single dose of 100 kBq/kg, statistical significance was achieved when compared with the vehicle control (*P* < 0.001). However, the PARPi combination was not significantly different from the 100 kBq/kg monotherapy treatment. This lack of in vivo synergy may serve to highlight that inhibition of single-strand-break repair, mediated by PARP, has a less pronounced effect than targeting of DSB repair and the cell cycle checkpoint, mediated by ATR, in the response to α-radiation–induced DNA damage. In contrast, MSLN-TTC combined with ATRi (40 mg/kg) at a single dose of 100 kBq/kg resulted in a more pronounced tumor growth inhibition than did the combination with PARPi. The combination induced significant (*P* < 0.001) tumor growth inhibition, with the level of this effect being equivalent to the single dose of 250 kBq/kg of MSLN-TTC, and was determined to be strongly synergistic by the Bliss additivity model. This finding is in alignment with our observations from the in vitro studies and further supports the hypothesis that blockage of the DNA DSB repair machinery results in stronger therapeutic efficacy. Further studies need to be performed in order to investigate the effect of alternative dosing schedules.

A second human ovarian cancer xenograft model using the OVCAR-8 cell line was selected for further evaluation of the ATRi combination. This model had a lower level of receptors and a more rapid growth rate. The dosing regimen was therefore changed to 3 × 200 kBq/kg administered as monotherapy or in combination with ATRi (40 mg/kg dosed twice daily, 2 d on/5 d off). Each single agent (MSLN-TTC or ATRi) induced a significant tumor growth inhibition (*P* < 0.0001) compared with vehicle. However, the combination resulted in a significant increase of tumor growth inhibition compared with both monotherapies (*P* < 0.001), demonstrating the synergistic activity. All doses were well tolerated as evidenced by no critical body weight loss. Hematologic analysis showed comparable values of MSLN-TTC monotreatment and the combination with ATRi, indicating that there was no increased toxicity from the combination treatment.

In summary, these findings demonstrate that the combination effect appears to be DDR pathway–dependent. The ATRi has been reported to induce synthetic lethality in tumors with defects in G1 cell cycle checkpoint, including mutations in ATM and TP53 (25). Interestingly, the monotherapy with ATRi was more efficacious in the OVCAR-8 model (ATM^mut^/TP53^mut^), inducing a significant tumor growth inhibition in comparison to the OVCAR-3 model (TP53^mut^) (27). The mutations may have contributed to the enhanced efficacy of the ATRi treatment in the OVCAR-8 model, as ATM defects have been described to make cells more dependent on ATR. However, as these models are not sufficiently comparable, a broader assessment of the therapeutic relevance of targeting specific mutations in relation to synergy in preclinical models will be the subject of future work. It also remains to be explored if the synergy of MSLN-TTC/PARPi combinations can be enhanced in tumors characterized by BRCA1/2 defects, as the PARP/BRCA combination is well described to induce synthetic lethality (28).

## CONCLUSION

The presented study supports the rationale for combining the MSLN-TTCs with DDR inhibitors based on their individual mode of action as a new strategy for treating ovarian cancer characterized by overexpression of MSLN.

## DISCLOSURE

The Research Council of Norway funded this study. All authors are employees of Bayer AS or Bayer AG. The work was included in a submitted patent application. No other potential conflict of interest relevant to this article was reported.

## KEY POINTS

**QUESTION:** Does blockage of DNA damage response (DDR) increase sensitivity to targeted α-therapy (TAT)?
**PERTINENT FINDINGS:** A statistically significant synergistic effect was observed both in vitro and in vivo on combination of the TAT mesothelin-targeted ^227^Th conjugate with an ATR or PARP inhibitor.
**IMPLICATIONS FOR PATIENT CARE:** These data support the mechanism-based clinical potential of combining TAT with DDR inhibitors with the aim of enhancing response rates in patients.
