# Supplementary material for: Synergistic Effect of a Mesothelin-Targeted 227Th Conjugate in Combination with DNA Damage Response Inhibitors in Ovarian Cancer Xenograft Models
Source: J Nucl Med. 2019 Sep;60(9):1293–300. doi: 10.2967/jnumed.118.223701 (PMC6735281; doi:10.2967/jnumed.118.223701)
Supplement: Supplementary file 1 [file jnm223701SupplementalData.pdf]

Supplemental Figure 1: FACS Analysis Comparing Binding Potency of MSLN-antibody Conjugate

Cells were seeded in 96 well plates (100 000 cells, 100 µl) and incubated with a titration of 0.0006-100 µg/ml of anti-MSLN antibody, MSLN-antibody conjugate and isotype control for one hour at 4°C, followed by incubation with 100 µl anti-human IgG-PE (Cat# 409304, biolegend) for one hour at 4°C. The mean fluorescence intensity (MFI) was calculated using GraphPad Prims software version 7.0 and was plotted against the protein concentration. The mAbs/cell was determined making a standard curve using beads from Quantibrite (BD biosciences).

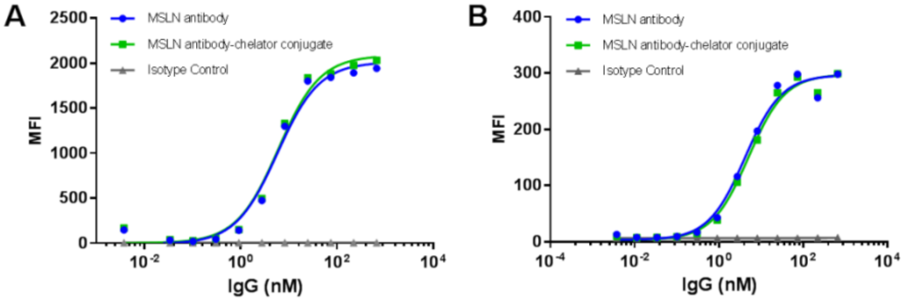

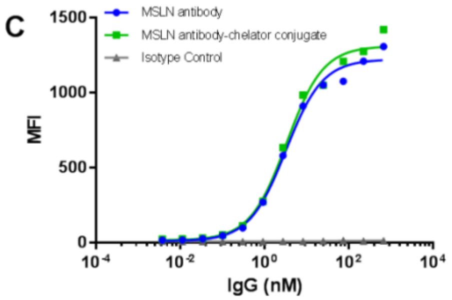

	MSLN antibody - chelator conjugate	MSLN antibody
EC_{50} (nM) HT29-MSLN	19	15
EC_{50} (nM) Ovcar-3	3.1	2.5
EC_{50} (nM) NCI-H226	3.2	3.3

Supplemental Figure 1. FACS analysis on different cell lines, comparing binding potency of naked MSLN antibody with MSLN-antibody conjugate. An isotype control conjugate was included to demonstrate specificity. Data were fitted using Graph Pad Prism software, EC50 values are presented in the table. Binding to **A)** HT29-MSLN cells; **B)** Ovcar-3 cells; **C)** NCI-H226 cells

Supplemental Figure 2: ELISA on recombinant human MSLN.

For ELISA, recombinant human MSLN was coated to 96-well plates (1 µg/mL; NUNC/Maxisorp). Wells were blocked with 3 % BSA in PBS. Cold MSLN-antibody conjugate, an isotype control antibody and the radiolabeled MSLN-TTC (7 MBq/10mg, stored for 72 hours) were titrated (1:3; 100 µg/mL) on the MSLN coated ELISA plate. Unbound samples were washed off and bound samples were visualized using horseradish peroxidase labeled goat anti-human lambda antibody (Southern Biotech) followed by visualization with the peroxidase substrate ABTS (Life Technologies). The absorbance was measured at 405 nm in a plate reader (Perkin Elmer). EC_{50} values were calculated using GraphPad Prism Software.

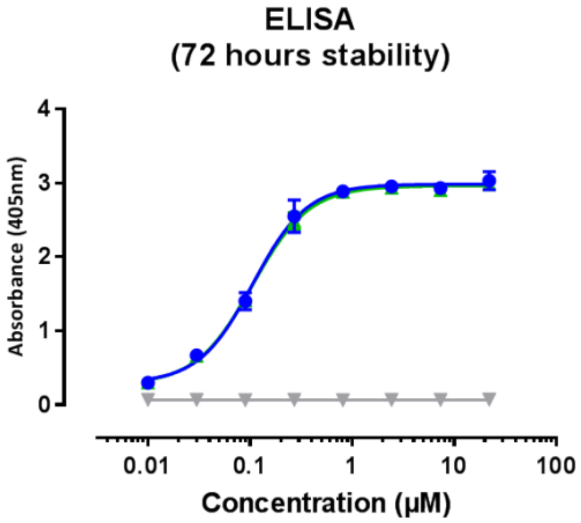

	MSLN antibody-chelator conjugate	MSLN-TTC
EC50 (µM)	0.1065	0.1054

Supplemental Figure 2. ELISA on recombinant human MSLN. Binding affinity of the radiolabeled MSLN-TTC (7 MBq/ 10 mg) is compared against the antibody-chelator conjugate and isotype control after 72 hours incubation, demonstrating no change in binding affinity.

Supplemental Figure 3: Isobologram Generated from CAPAN-2 Cell Line Treated with MSLN-TTC in Combination with DDR Inhibitors.

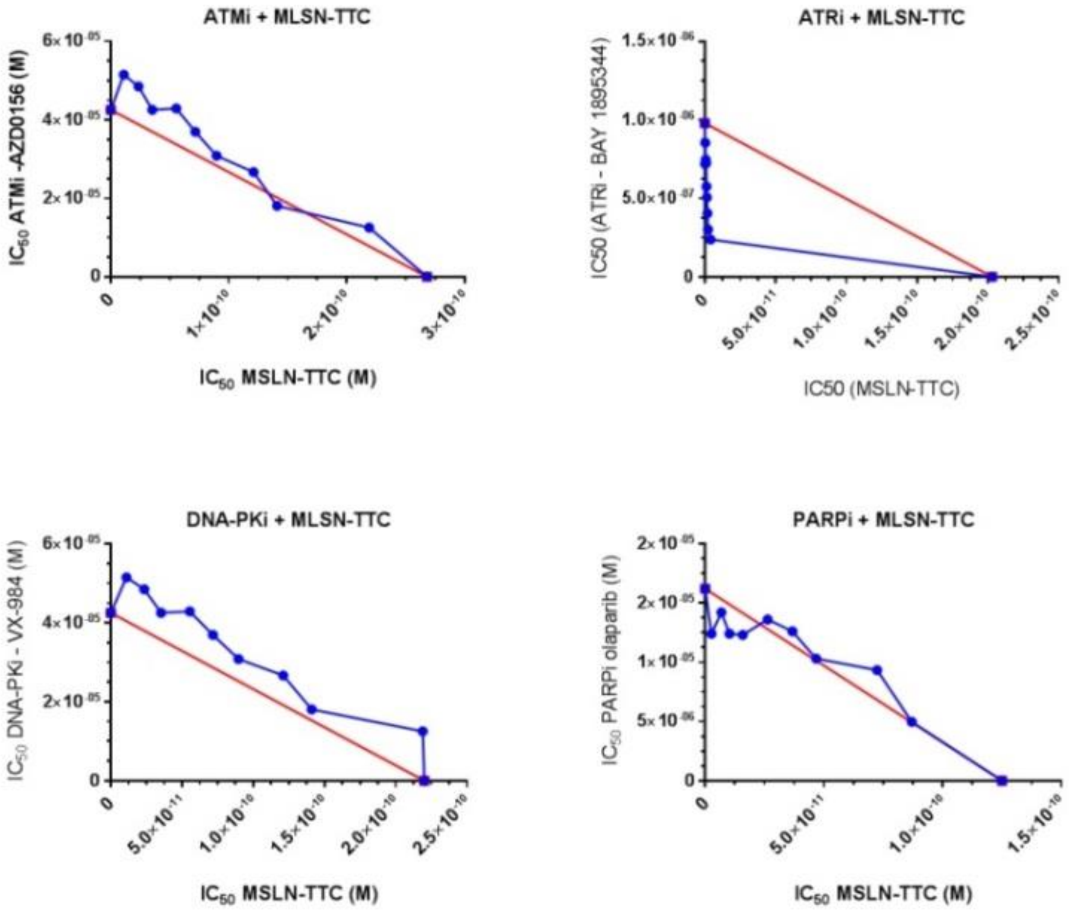

Supplemental Figure 3. Isobologram generated from CAPAN-2 cell line treated with MSLN-TTC in combination with DDR inhibitors. Cell viability was determined by use of CellTiterGlo. The IC_{50}-isobolograms were generated by plotting the actual IC_{50} values of MSLN-TTC and DDRi along the x- and y-axis, respectively.

Supplemental Figure 4: Isobologram generated from HT29-MSLN Cell Line Treated with MSLN-TTC in Combination with DDR Inhibitors.

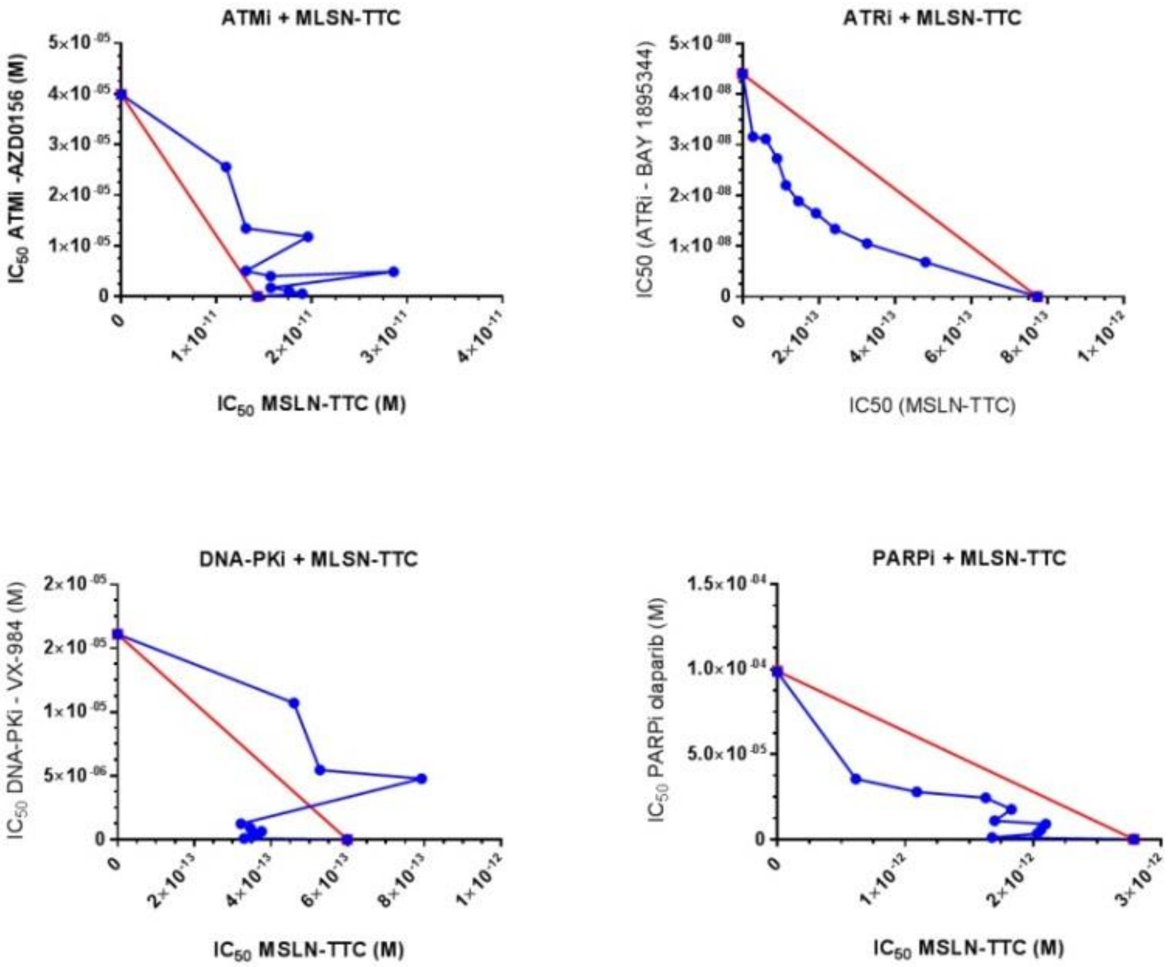

Supplemental Figure 4. Isobologram generated from HT29-MSLN cell line treated with MSLN-TTC in combination with DDR inhibitors. Cell viability was determined by use of CellTiterGlo. The IC_{50}-isobolograms were generated by plotting the actual IC_{50} values of MSLN-TTC and DDRi along the x- and y-axis, respectively.

Supplemental Figure 5: **Isobologram generated from NCI-H226 Cell Line Treated with MSLN-TTC in Combination with DDR Inhibitors.**

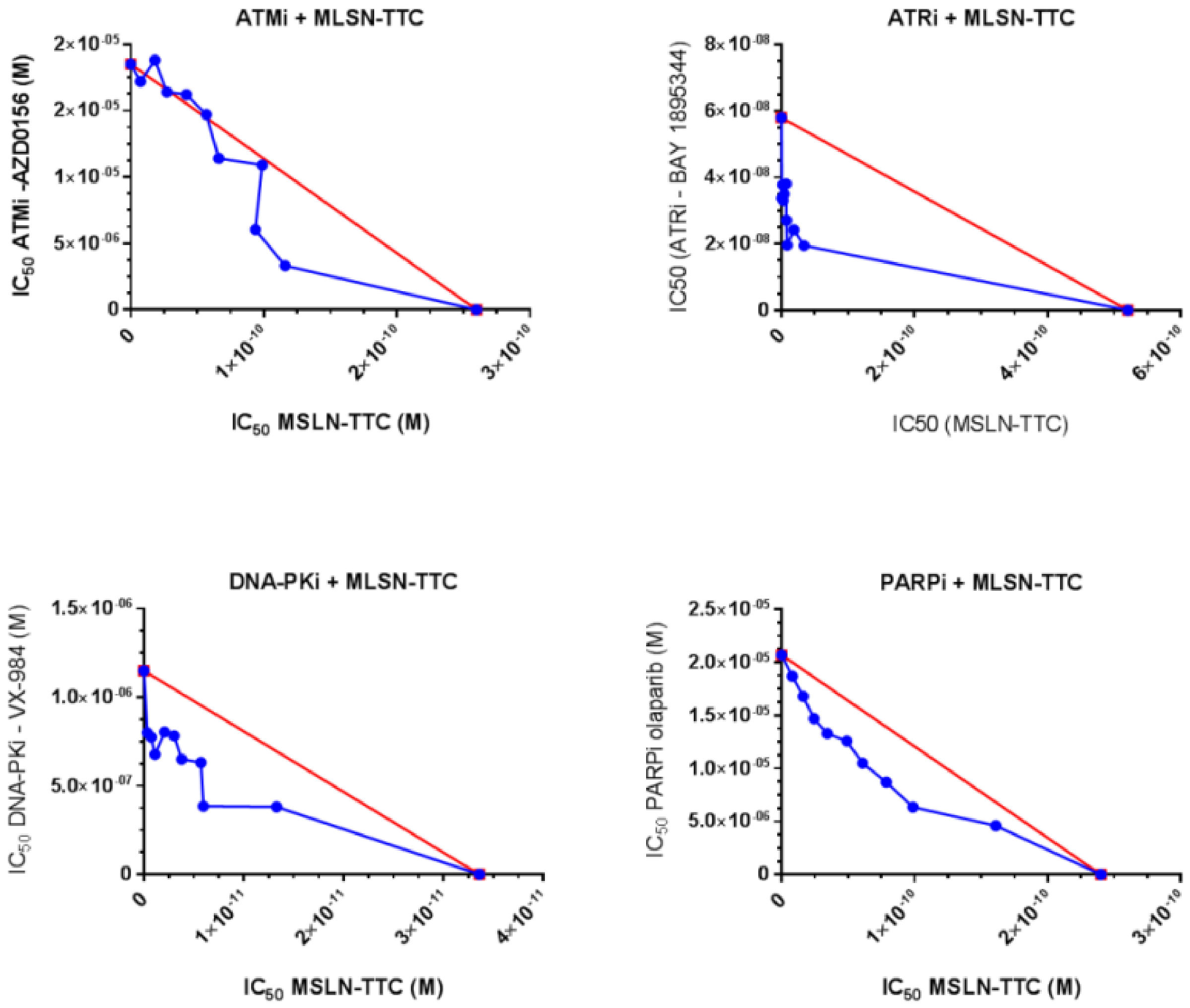

Supplemental Figure 5. **Isobologram generated from NCI-H226 cell line treated with MSLN-TTC in combination with DDR inhibitors.** Cell viability was determined by use of CellTiterGlo. The IC_{50}-isobolograms were generated by plotting the actual IC_{50} values of MSLN-TTC and DDRi along the x- and y-axis, respectively.

Supplemental Figure 6: Isobologram generated from OVCAR-8 Cell Line Treated with MSLN-TTC in Combination with ATR Inhibitor.

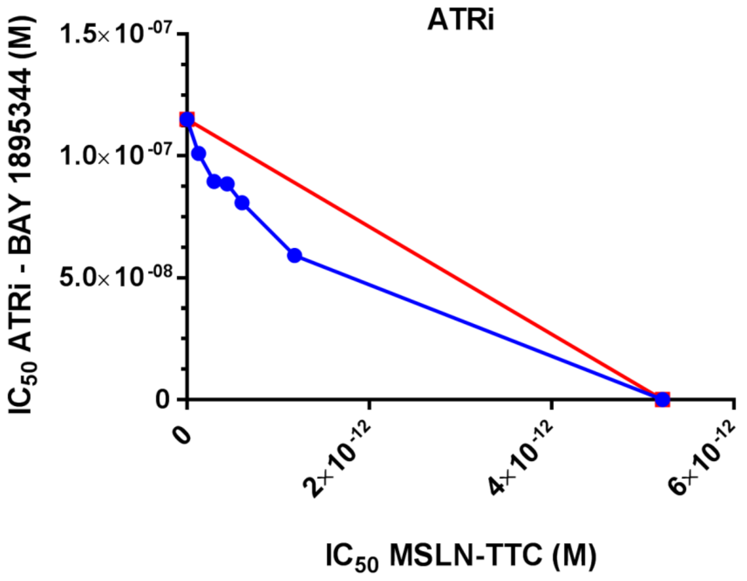

Supplemental Figure 6. Isobologram OVCAR-8 treated with MSLN-TTC in combination with ATRi. Cell viability was determined by use of CellTiterGlo. The IC_{50}-isobolograms were generated by plotting the actual IC_{50} values of MSLN-TTC and DDRi along the x- and y-axis, respectively.

Supplemental Figure 7. *In Vitro* Experiments from MSLN-TTC +/- ATRi BAY 1895344 or PARPi olaparib on OVCAR-3

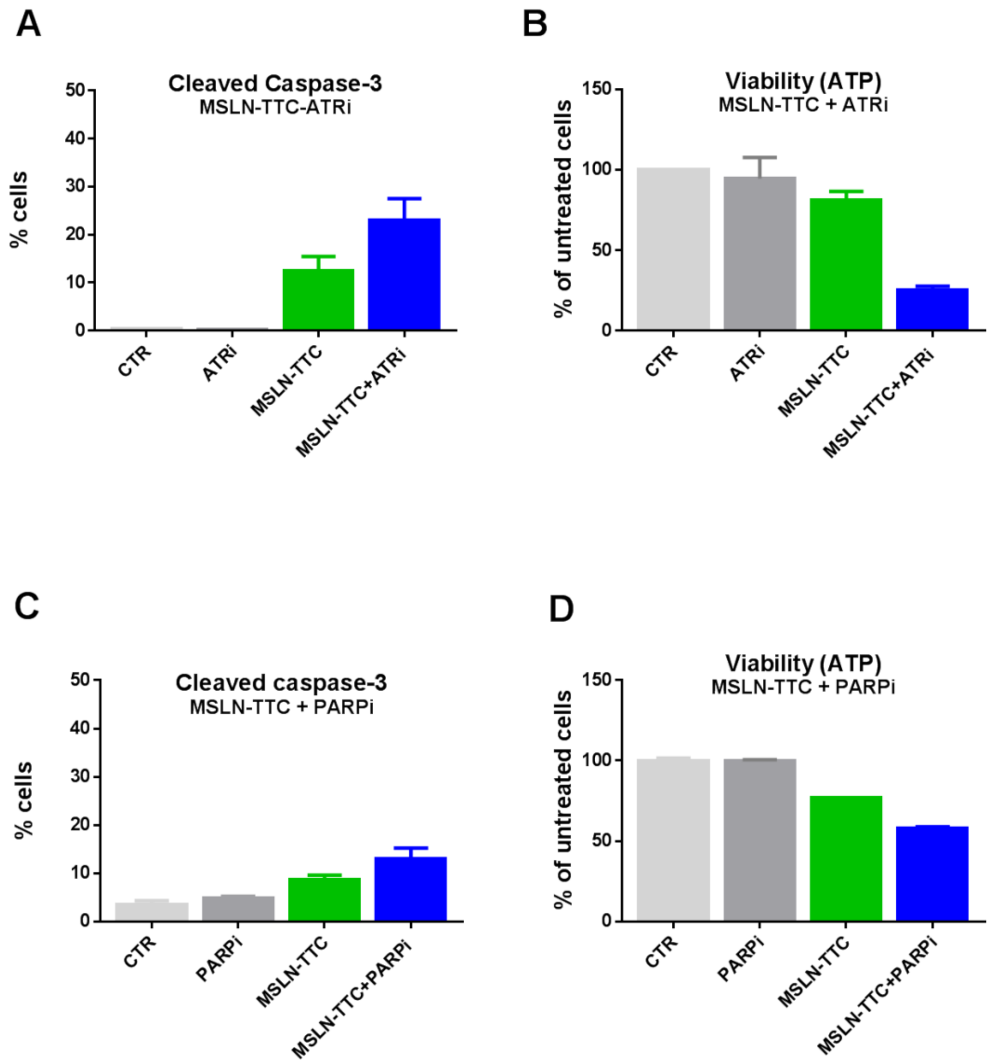

Supplemental Figure 7. *In vitro* experiments from MSLN-TTC +/- ATRi BAY 1895344 or PARPi olaparib on OVCAR-3. A-B) Apoptosis and viability determination after combination treatment with MSLN-TTC (10 kBq/ml) and BAY 1895344 (10 nM). C-D) Apoptosis and viability determination after combination treatment with MSLN-TTC (1 kBq/ml) and olaparib (0.5 µM).

Supplemental Tables 1 and 2

Table 1: Mean Values ± SD of Mechanistic Markers MSLN-TTC + ATRi

Marker	CTR	MSLN-TTC	ATRi	MSLN-TTC + ATRi
DSB (γH2A.X)	5.3 ± 1.0	26.3 ± 2.1	6.3 ± 0.5	43.3 ± 1.8
Apoptosis (Cleaved Caspase-3)	0.4 ± 0.3	12.45 ± 3.0	0.3 ± 0.3	23.0 ± 4.5
Viability (ATP)	100 ± 0.5	81.1 ± 5.5	94.5 ± 2.4	25.3 ± 2.4

Table 2: Mean Values ± SD of Mechanistic Markers MSLN-TTC + olaparib

Marker	CTR	MSLN-TTC	olaparib	MSLN-TTC + olaparib
DSB (γH2A.X)	4.6 ± 1.2	29.6 ± 0.7	10.8 ± 0.4	35.8 ± 3.5
Apoptosis (Cleaved Caspase-3)	3.5 ± 0.8	8.7 ± 1.0	4.9 ± 0.4	13.1 ± 2.1
Viability (ATP)	100 ± 1.5	76.9 ± 0.2	99.8 ± 0.8	57.8 ± 1.3

Supplemental Figure 8: Body Weights Determined after Treatment with MSLN-TTC in Combination with ATRi BAY 1895344 or PARPi olaparib.

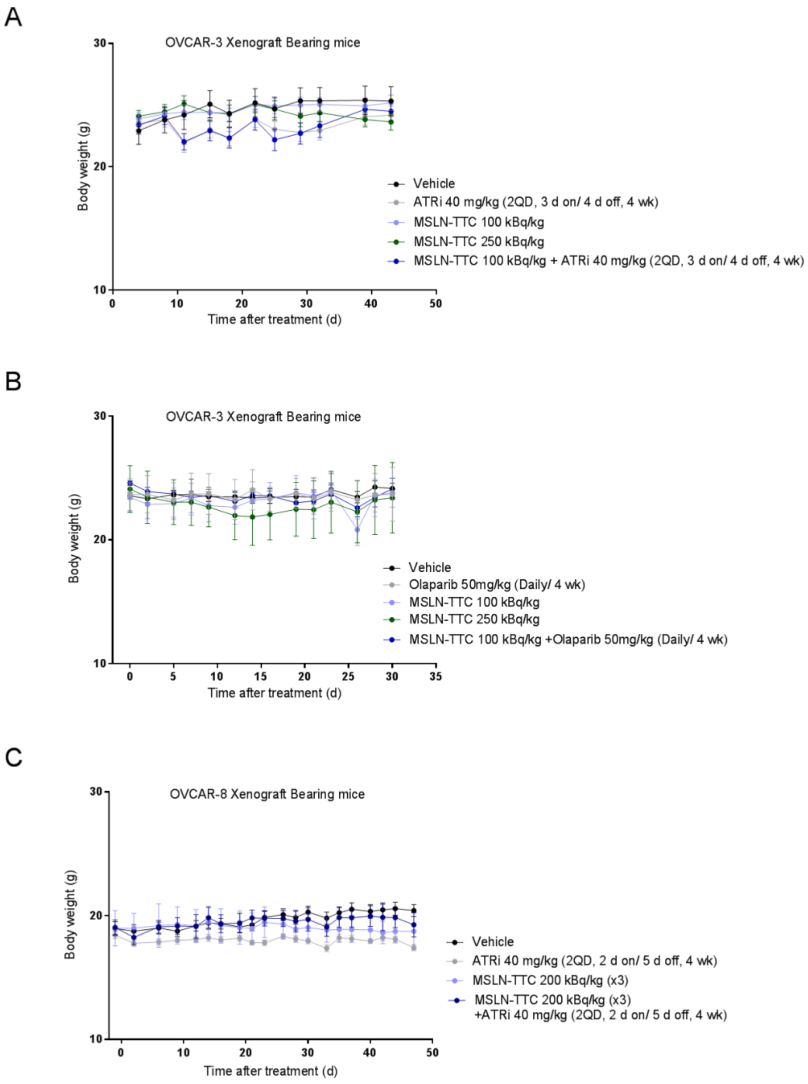

Supplemental Figure 8. Body weights determined after treatment with MSLN-TTC in combination with ATRi BAY 1895344 or PARPi olaparib. A) Body weight of OVCAR-3 xenograft bearing mice determined after a single dose administration of MSLN-TTC (100 kBq/kg, 0.14 mg/kg, i.v.) and ATRi (40 mg/kg 2QD, 3 days on/ 4 days off, 4 weeks), B) Body weight of OVCAR-3 xenograft bearing mice determined after a single dose administration of MSLN-TTC (100 kBq/kg, 0.14 mg/kg, i.v.) and olaparib (50 mg/kg QD for 4 weeks), C) Body weight of OVCAR-8 xenograft bearing mice determined after three intravenous (i.v.) injections of MSLN-TTC (200 kBq/kg, 0.14

mg/kg, day 1, 22 and 43) and BAY 1895344 (40 mg/kg 2QD, 2 days/5 days off, 7 weeks).
